# Identification and Functional Characterization of Mutation in *FYCO1* in Families with Congenital Cataract

**DOI:** 10.3390/life13081788

**Published:** 2023-08-21

**Authors:** Muhammad Ikram Ullah, Zaira Rehman, Rubina Dad, Abdullah Alsrhani, Muhammad Shakil, Heba Bassiony Ghanem, Ayman Ali Mohammed Alameen, Mohamed Farouk Elsadek, Lienda Bashier Eltayeb, Sajjad Ullah, Muhammad Atif

**Affiliations:** 1 Department of Clinical Laboratory Sciences, College of Applied Medical Sciences, Jouf University, Sakaka 72388, Saudi Arabia; afalserhani@ju.edu.sa (A.A.); hbghanem@ju.edu.sa (H.B.G.); aaalameen@ju.edu.sa (A.A.M.A.); maahmad@ju.edu.sa (M.A.); 2 Department of Pathology, Indus Hospital & Health Network, Karachi 75190, Pakistan; zaira.rehman@tih.org.pk; 3 Structure Biology Research Centre, Human Technopole, 20157 Milan, Italy; 4 Department of Biochemistry, King Edward Medical University, Lahore 54600, Pakistan; mshakil@kemu.edu.pk; 5 Department of Biochemistry, University of Health Sciences, Lahore 54600, Pakistan; 6 Department of Community Health Sciences, College of Applied Medical Sciences, King Saud University, Riyadh 11433, Saudi Arabia; mfbadr@ksu.edu.sa; 7 Department of Medical Laboratory Sciences, College of Applied Medical Sciences, Prince Sattam Bin Abdul-Aziz University, Al-Kharj, Riyadh 11942, Saudi Arabia; l.eltayeb@psau.edu.sa; 8 University Institute of Medical Laboratory Technology, Faculty of Allied Health Sciences, The University of Lahore, Lahore 54600, Pakistan; sajjad.ullah@mlt.uol.edu.pk

**Keywords:** congenital cataract, *FYCO1*, mutations, consanguineous families, in silico analysis

## Abstract

Congenital cataract (CC) causes a third of the cases of treatable childhood blindness worldwide. CC is a disorder of the crystalline lens which is established as clinically divergent and has complex heterogeneity. This study aimed to determine the genetic basis of CC. Whole blood was obtained from four consanguineous families with CC. Genomic DNA was extracted from the blood, and the combination of targeted and Sanger sequencing was used to identify the causative gene. The mutations detected were analyzed in silico for structural and protein–protein interactions to predict their impact on protein activities. The sequencing found a known *FYCO1* mutation (c.2206C>T; p.Gln736Term) in autosomal recessive mode in families with CC. Co-segregation analysis showed affected individuals as homozygous and carriers as heterozygous for the mutation and the unaffected as wild-type. Bioinformatics tools uncovered the loss of the Znf domain and structural compactness of the mutant protein. In conclusion, a previously reported nonsense mutation was identified in four consanguineous families with CC. Structural analysis predicted the protein as disordered and coordinated with other structural proteins. The autophagy process was found to be significant for the development of the lens and maintenance of its transparency. The identification of these markers expands the scientific knowledge of CC; the future goal should be to understand the mechanism of disease severity. Ascertaining the genetic etiology of CC in a family member facilitates establishing a molecular diagnosis, unlocks the prospect of prenatal diagnosis in pregnancies, and guides the successive generations by genetic counseling.

## 1. Introduction

Congenital cataract (CC) or pediatric cataract is one of the most challenging eye disorders which can lead to severe vision loss and childhood blindness globally. Its diagnosis can be established at birth or during the first year of life [1]. It can be congenital or acquired, unilateral or bilateral, and is highly treatable. A cataract is a primary event of vision impairment and total blindness, and CC causes a third of total blindness cases all around [2,3]. Therefore, it is crucial to screen children for the condition during neonatal life. Due to the reduced reflex index and low lens clarity, the characteristic symptoms of CC include blurred vision, photophobia, double vision, and nyctalopia [4]. CC can damage the nerves of the visual development pathway resulting in visual deprivation and causes irreversible bilateral amblyopia [5,6]. In addition, CC elicits changes in lens architecture or structure that lead to opacity of the ocular lens or cloudy vision [7].

Developmental cataract (after 1 year of age) has a prevalence of 0.32 to 22.9 cases in 10,000, while congenital cataract (present from birth) has a prevalence of 0.63 to 9.74 per 10,000 [3], although the highest incidence rate of CC is observed in Asia. The various causes of CC include idiopathic (62.2%), inherited (22.3%), and non-hereditary (11.5%) [2]. Also, it has been reported that isolated CC accounted for 62.3%, CC with ocular disorders rated 22.7%, and CC with systemic disorders recorded in 17.3% of cases. On the other hand, unilateral CC constitutes 56% of idiopathic CC but just 6% of hereditary CC, whereas bilateral CC constitutes 56% of hereditary cases [2,7]. Cataracts account for almost 39% of cases of blindness globally, but it is reported as occurring at a higher frequency (51%) in the Pakistani population because of its higher rates of consanguinity. The frequency of CC is about 23% in Pakistani families from the total of 54.7% of visually handicapped children in this population [8,9].

CC is categorized into a variety of subgroups pertaining to their phenotypic–genotypic characteristics. Based on the localization and morphology of opacities, these CC subtypes are classified as nuclear, lamellar, anterior and posterior polar, cortical, and total cataracts [8]. CC shows complex heterogeneity because it is highly divergent with variable inheritance patterns [10]. Although the prevalent inheritance pattern of CC is autosomal dominant, it can be autosomal recessive or X-linked in rare cases [11,12]. In 50-63% of CC, the etiology is idiopathic, while 30% are monogenic with autosomal dominant pattern [12]. To date, more than 1400 etiologic variations have been described for this disease [11,13,14,15,16]. Non-syndromic cataract is linked to 39 causative genes or loci. Autosomal recessive CC has been mapped to 14 loci, but the genes for some loci have not been identified yet [14]. Several causative genes of CC include *EPHA2* (OMIM 176946), *GJA8* (OMIM 600897)*, FOXE* (OMIM 601094)*, FYCO1* (OMIM 607182)*, GCNT2* (OMIM 600429)*, AGK* (OMIM 610345)*, AKR1E2* (OMIM 617451)*, RNLS* (OMIM 609360)*, HSF4* (OMIM 602438)*, LIM2* (OMIM 154045)*, CRYBA1* (OMIM 123610)*, LSS* (OMIM 600909)*, CRYBB3* (OMIM 123630)*,* and *GALK1* (OMIM 604313) [11]. *FYCO1* is the most frequent genotype of CC in Pakistani families [8,11].

The FYVE and coiled-coil domain-containing 1 (*FYCO1*) gene, localized on the physical map at 3p21.31, contains 18 coding exons (NM_024513.4) and plays a vital role in the development and transparency of the lens in humans [17]. Moreover, FYCO1 protein is elucidated as an autophagy adaptor protein and a constituent of the phosphatidylinositol 3-phosphate (PIP3)-binding protein family [18]. Several previous studies showed that autosomal recessive CC could be related to variations in the *FYCO1* gene. The mutations associated with *FYCO1* are the most frequent in Pakistani families with cataracts and contribute about 14% to the total genetic load in this population. The common nonsense mutation p.Gln736Ter (Q736X) has been reported in Egyptian, Iranian, and Pakistani children with CC [13,16,19].

During the process of autophagy, FYCO1, an adaptor protein, promotes microtubule plus-end-directed autophagosomes through instantaneous interaction with kinesin motor proteins and autophagosomal membrane components such as RAB7 and microtubule-associated protein 1 light chain 3 (LC3) PI3P [20,21].

This study was conducted to detect causative genes in complex families with CC and to predict the structural and conformational impacts of the mutation on protein activity.

## 2. Materials and Methods

The institutional review board of the University of Health Sciences, Lahore, Pakistan, granted ethical approval for this study before its commencement. The study applied the updated Helsinki guidelines (2013) for inclusion of human subjects. Informed consent was obtained from family members or their guardians.

### 2.1. Population Recruitment

A total of four families with CC were recruited from the Sindh Province of Pakistan following written informed consent. Pedigrees were drawn, and whole blood was collected from affected and unaffected family members.

### 2.2. Molecular Studies

Genomic DNA was extracted from the whole blood according to the protocol described in a previous study [22]. The DNA of one affected member of each of the families recruited was subjected to targeted sequencing, including for variants or genes linked to all syndromic and non-syndromic cataracts. Then, co-segregation was confirmed by Sanger sequencing of all putative variants of *FYCO1* in the families studied. All wild-type data were retrieved and aligned with the human genome reference sequence (hg19.0) using BioEdit 7.0 and Chromas 2.5 software (Informer Technologies, Inc., Los Angeles, CA, USA) to detect variations in sequencing data.

### 2.3. Bioinformatics Analysis of FYCO1 Mutations

#### 2.3.1. Primary Sequence Analysis of FYCO1

##### Protein Order–Disorder Prediction

To determine the order–disorder pattern of the FYCO1 protein, the web-based tools Meta Disorder [23] and MobiDB [24] were utilized. Thirteen tools implemented in Meta Disorder predict the disorder regions of the protein by employing six tools (GlobPlot, DisEMBL, IUPred, ESpritz, VSL2b, and Jronn). The type of disordered FYCO1 was studied using the PONDR server to identify protein location by charge–hydropathy plot and by applying the cumulative distribution function formula [25]. Disordered proteins carry hydrophilic amino acids and have varied arrangements. Composition Profiler was applied to determine the hydrophobicity and flexibility of amino acid composition (http://www.cprofiler.org/, accessed on 17 January 2023).

##### Analysis of Protein Conservation

The analysis of amino acid conservation was performed through the ConSurf server. It determines the evolutionary conservation of amino acids based on phylogeny [26]. It uses 150 sequence homologues for analysis to achieve 95% confidence. The Bayesian method was used to calculate final scores.

##### Prediction of Physicochemical Properties of FYCO1

The primary sequence of FYCO1 was analyzed to ascertain its accessibility, mutability, polarity, and bulkiness properties by using the ProtScale server accessed from the platform on Expasy (http://web.expasy.org/protscale/, accessed on 3 February 2023). Cleavage site and signal peptide prediction was performed by SignaIP-4.1 (http://www.cbs.dtu.dk/services/SignalP/, accessed on 13 March 2023) based on an artificial neural network, and Netphos 3.1 server (http://www.cbs.dtu.dk/services/NetPhos, accessed on 20 March 2023) determined the phosphorylation sites for each Thr, Ser, and Tyr residue with a 0.5 threshold. The Protparam server (http://web.expasy.org/protparam/, accessed on 8 April 2023) estimated the half-life, molecular weight, and amino acid composition of the FYCO1 protein.

##### Prediction of the Secondary Structure of FYCO1

The secondary structure of FYCO1 was studied using a web-based GOR4 server for gathering 3-D information on protein [27] and the JPred3 web-based server for predicting the secondary structure of protein (alpha helix, beta strand, and coil) [28].

#### 2.3.2. Tertiary Structure Prediction

We applied BLAST to search for the amino acid sequence of FYCO1 and did not find any homology with the crystal structures in the protein data bank. Therefore, without a structural homologue, the ab initio modeling approach was employed using trRosetta [29]. Five models were generated, which were then evaluated using a Ramachandran plot.

#### 2.3.3. Protein–Protein Interaction Analysis of FYCO1

For other cellular protein interaction studies of FYCO1, the String database (https://string-db.org/, accessed on 12 April 2023) was used for previously known interactions or to predict naive protein–protein interactions. To determine interactive partners of FYCO1, a confidence interval (CI) was applied for the first shell with 20 interactions and for the second shell with 10 interactions; values were calculated by setting a median of 0.4. The scores can directly or indirectly indicate physical and functional interactions [30].

#### 2.3.4. Domain-Dependent Mutation Analysis of FYCO1

A literature search for all known cataract-causing mutations in FYCO1 was performed based on domain-wise locations and mutation type.

## 3. Results

### 3.1. Molecular Studies

**Figure 1:**
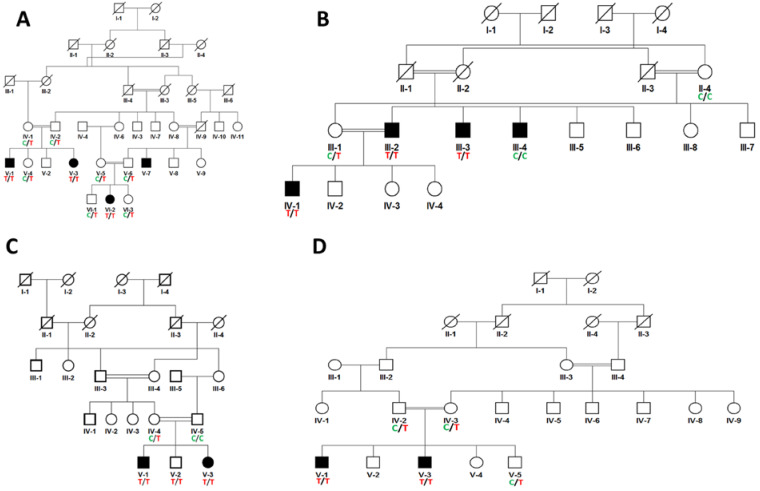
Pedigree analysis of consanguineous families (**A**–**D**) shows the autosomal recessive mode; filled circles and squares represent affected members and empty circles and squares represent unaffected individuals; vertical lines are generation lines and horizontal lines represent siblings. In pedigrees, the genotype C/C indicates the wild-type homozygous pattern in unaffected individuals, C/T genotypes show a heterozygous pattern in carrier individuals, and the genotype T/T represents a homozygous pattern in affected individuals.

**Figure 2:**
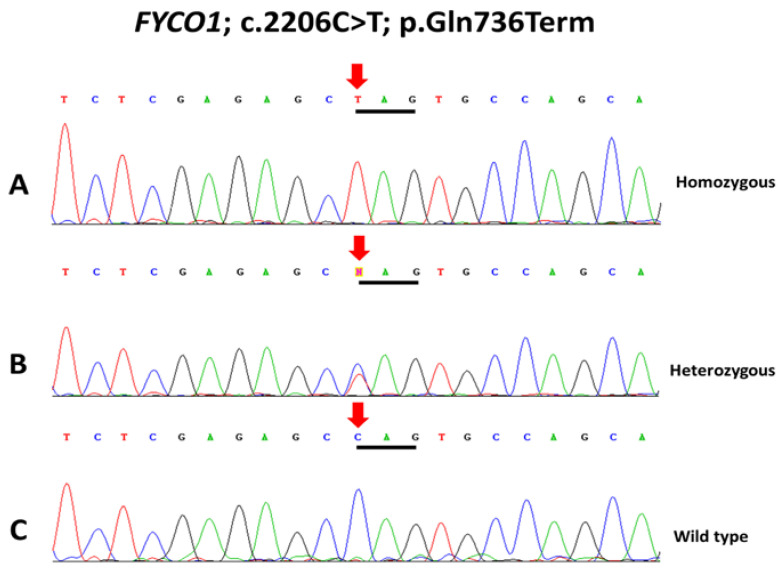
Sequencing data of four families with congenital cataract show that affected individuals are homozygous (**A**) and carriers (parents) are heterozygous (**B**) for *FYCO1*; c.2206C>T; p.Gln736Term, whereas unaffected members are homozygous wild-type (**C**).

This study includes four families with congenital cataracts, and pedigree analysis-inferred autosomal recessive inheritance pattern (Figure 1). Targeted sequencing identified a known nonsense mutation, *FYCO1*; c.2206C>T; p.Gln736Term, in all four families (Figure 2). This variant results in truncated or premature protein; hence it is pathogenic according to ACMG classification. Co-segregation analysis identified all carriers as heterozygous, normal individuals with wild-type sequences, and affected individuals as homozygous for the nucleotide variation.

### 3.2. Bioinformatics Analysis

#### 3.2.1. Primary Structure Analysis of FYCO1

##### Prediction of FYCO1 Order/Disorder Patterns

**Figure 3:**
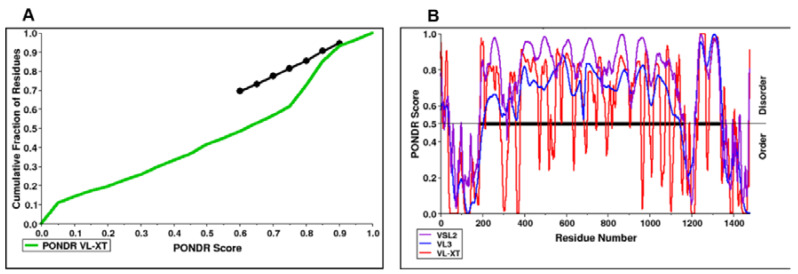
Disorder prediction of FYCO1. (**A**) CDF plot from the PONDR server that characterizes the protein as an order of disorder. The reference line is shown in black; the predicted order proteins are shown above the line and disorder proteins below the line. The green line under the black line represents a disordered protein with only a few ordered regions (touching the black line at a point). (**B**) The amino acid distribution in terms of order/disorder. A 0.5 score is considered a reference-line fpr protein; the protein is disordered above the line, while it is ordered below the line.

**Figure 4:**
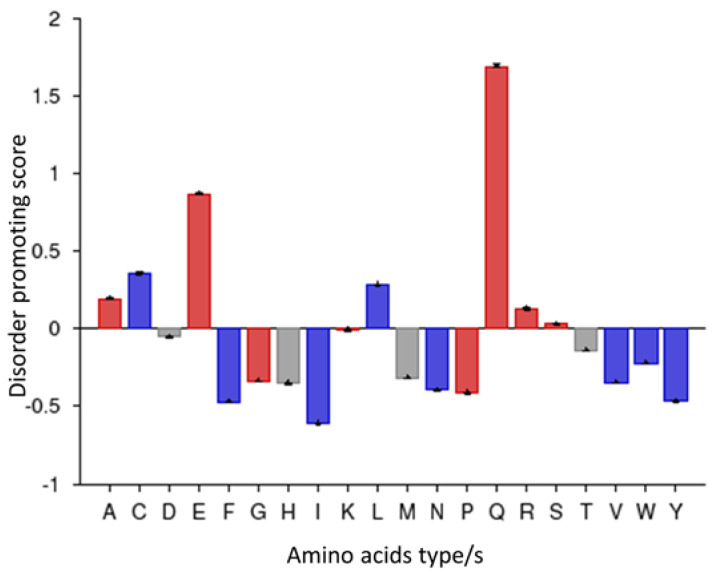
Disorder-promoting amino acids of the FYCO1 protein. The blue color represents the order-promoting and the red color represents disorder-promoting amino acids. Neutral amino acids are shown in gray.

**Figure 5:**
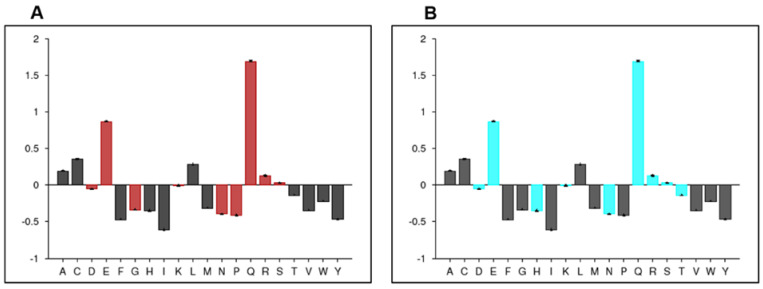
(**A**) Flexibility of amino acid composition of FYCO1. The black color represents amino acid rigidity, while the red color indicates flexible amino acids. (**B**) Hydrophobicity of FYCO1. The cyan color indicates hydrophilic amino acids and the black color indicates hydrophobic amino acids.

According to the PONDR server prediction, the FYCO1 protein is a mixture of order and disorder regions. A region almost 100 amino acids long at the N-terminal and another region of almost 300 amino acids at the C-terminal were found to be ordered, while the rest of the protein was disordered (Figure 3). The protein was further characterized based on contributory amino acids—whether or not they were order-promoting. The amino acid composition profile of the protein showed that it begins development with disorder-promoting amino acids such as glutamine and glutamic acid (Figure 4). FYCO1 is enriched with flexible amino acids, which again favor the disordered behavior of the protein (Figure 5A). The protein is also enriched with hydrophilic amino acids while hydrophobic amino acids are depleted (Figure 5B).

##### Protein Conservation Analysis

The ConSurf analysis showed that Q736 has exposed residues with an average conservation score (Supplementary Figure S1). The degree of conservation was identified on the basis of evolutionary changes by performing multiple sequence alignments.

##### Physicochemical Properties of FYCO1

The Protscale server was used to determine the physicochemical properties of FYCO1 (Table 1). A score higher than the cutoff value indicated a higher probability of precision of FYCO1 features (Supplementary Figure S2). Protein bulkiness was calculated as the ratio of the side chain amino acid volume to its length. It affects the crystalline material of a particular protein. The values for FYCO1 fell within the range of 8.7 (aa position 1239) to 19.167 (aa position 1350). The Hopp–Woods scores predicted hydrophobicity, which ranged between −1.333 (1206 aa) and 2.122 (aa 415).

This study also predicted Zimmerman’s score to show the polarity of FYCO1 through dipole–dipole intermolecular interactions between positively charged and negatively charged particles. The expected score lays between 0.390 (aa 974) and 39.432 (aa position 415). These results suggest the possible polarity of FYCO1. The probability of mutability, i.e., amino acid conservation changes, during evolution was also determined for FYCO1 based on the relative score for mutability, which ranged from 43.667 (aa position 1202–1203) to 106.667 (aa position 5), suggesting the mutability potential of FYCO1. The SignalP server was used to compute the signal peptide of FYCO1. Different scores like the C-score (raw cleavage site score), S-score (signal peptide score: possible estimation of signal peptide), and Y-score (combined cleavage site score) were used to predict the signal peptide. The mean S-score and maximum Y-score can be used to calculate the average D-score to discriminate between non-signal peptides and signal peptides. The D-score of FYCO1 fell within a value of 0.0003, which is less than the cutoff value of 0.5; therefore, no signal peptide was predicted.

##### Predicted Phosphorylation Sites of FYCO1

**Figure 6:**
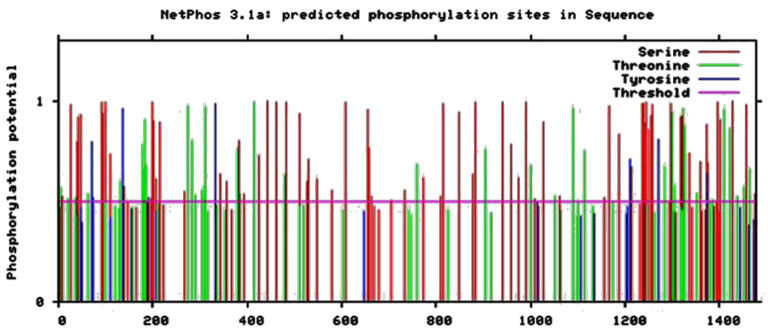
Netphos-predicted sites of potential phosphorylation in FYCO1.

We used the Netphos 3.1 server to predict potential phosphorylation sites of FYCO1. The server results showed that FYCO1 has ninety-two serine phosphorylation sites, thirty-nine threonine phosphorylation sites, and six sites of tyrosine-specific phosphorylation (Figure 6).

**Figure 7:**
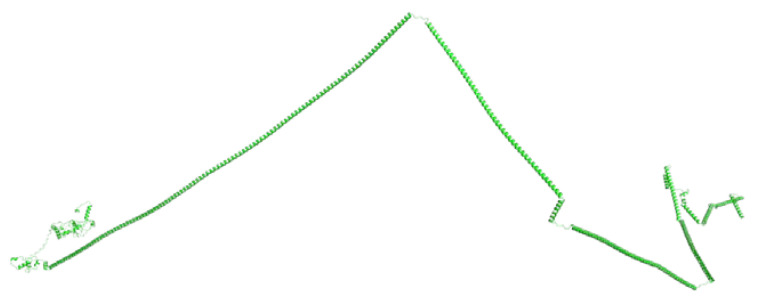
Three-dimensional structure of FYCO1 determined using trRosetta.

The *x*-axis of Figure 7 shows the N-terminal to C-terminal sequence of amino acids, whereas the *y*-axis represents the position of amino acids. The threshold score was ≤0.5 and the amino acid that crossed the threshold level showed potential phosphorylation.

### 3.3. Prediction of the Three-Dimensional Structure of FYCO1

A suitable template for homology modelling is lacking; therefore, the structure of FYCO1 was determined with ab initio modelling using trRosetta. Five models were generated and evaluated using a Ramachandran plot. The best model, with 98.5% of residues in the allowed region and only one residue in the disallowed region, was selected. The three-dimensional model suggested FYCO1 as a disordered protein, but with ordered N-terminal and C-terminal regions (Figure 7).

### 3.4. Analysis of Protein–Protein Interactions of FYCO1

**Figure 8:**
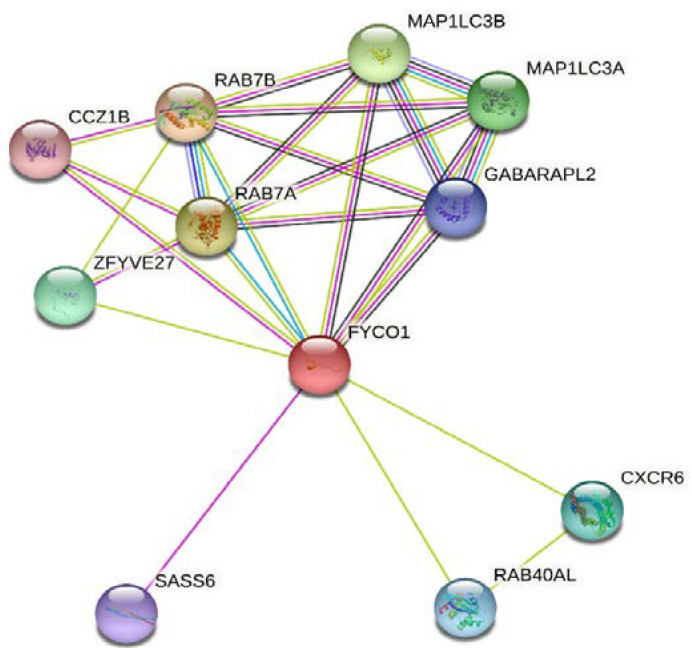
Protein–protein interaction network of FYCO1.

Analysis using STRING software 11.5 showed that FYCO1 has interaction potential with two members of the microtubule-associated protein 3 (MAP1LC3) family, MAP1LC3A and MAP1LC3B, both with functions in autophagy and autophagosome biogenesis (Figure 8). FYCO1’s other interaction was with RAB family members with functions in vesicle transport, such as RAB7B and RAB7A associated with Charcot–Marie–Tooth type 2 neuropathy and RAB40AL. FYCO1 also has interaction potential with CCZ1 homolog B (CCZ1B) involved in vacuolar transport, zinc finger FYVE-type containing 27 (ZFYVE27, the spastic paraplegia gene), C-X-C motif chemokine receptor 6 (CXCR6), and SAS-6 centriolar assembly protein (SASS6) involved in centriole duplication.

### 3.5. Domain-Dependent Mutation Analysis of FYCO1

**Figure 9:**
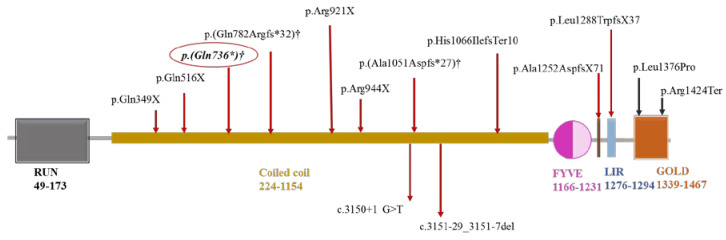
Domain-wise localization of all known mutations in FYCO1 causing cataract. The symbol * shows the stop codon at protein level, whereas † indicates the frequent protein mutant in coiled-coil domain.

We also analyzed all published mutations of FYCO1 causing cataracts. The mutations in different FYCO1 domains are illustrated in Figure 9. Out of a total of fourteen mutations, three are truncation mutations; ten mutations are present in the coiled-coil domain, two in the GOLD domain, one in the LIR domain, and one in between the FYVE and LIR domains of the currently unidentified domain.

## 4. Discussion

Congenital cataract is highly heterogeneous and is clinically and genetically complex. It is an increasingly prevalent pediatric anomaly that should be managed [31]. Etiological genetic investigations of inherited cataracts confirm that primary gene irregularities can be classified into four variant groups: variants comprising lens membrane protein; variants involving lens cytoskeletal elements; mutations in crystallin genes; and other enduring mutant types. Nevertheless, mutations in crystallin genes are more common. Generally, nuclear cataract has been associated with crystalline genes [6].

In the present study, a previously known mutation (c.2206C>T; p.Gln736Term) of the *FYCO1* gene was detected in four unrelated families with CC. This nonsense mutation resulted in a truncated protein on exon 8. The *FYCO1* gene is localized at physical position 3p21.31 on chromosome 3 with 17 coding and one non-coding exon. This gene encodes a protein that contains 1478 amino acids and has a mass of 166.9k daltons. Functional protein analysis found four domains in FYCO1: RUN domain with 36–173 amino acid residues; coiled-coil domain carrying a sequence of 224–1154 residues; Znf domain comprising a sequence of 1166–1231 residues; and GOLD domain with a sequence of 1339–1466 residues [14].

Several previous reports demonstrated that *FYCO1* genetic variants have been impeding in autosomal recessive form of CC. Until the present, out of the total reported mutations detected in *FYCO1* associated with CC, nineteen variants resulted in truncation of protein vulnerable with GOLD or coiled-coil protein domains [8]. In particular, an earlier study identified frameshift and nonsense mutation of *FYCO1* associated CC in thirteen unrelated families [14]. Additionally, another report determining the disease linked genetic abnormalities in three consanguineous families investigated one previously known and two naïve nucleotide variants in the *FYCO1* gene. It has been comprehended that nearly 15% of *FYCO1* genetic variants contributed to overall rates of autosomal recessive CC [8]. In near recent reports, *FYCO1* mutations detected in different ethnic groups include c.1387 G > T [13]; p.Gly463Ter; c.1621C > T [15]; p. Gln541Ter; and c.2365 C>T [14]. On the other hand, the largest exon 10 deletion and splice-site variant is c.3150 + 1G>T [14,16]. Some other known mutations were detected in Znf domain of FYCO1 protein including c.3150 + 1G>T; c.3151–2A>C; p.A1051DfsX27; c.3196delC; p.H1066IfsX10; and c.3670C>T; p.R1224X [14].

In this study, in silico tools predicted a Zimmerman score of 0.390 (aa 974) to 39.432 (aa position 415), suggesting the possible polarity of FYCO1 (mutant Q739X). The potential mutability of the conservation alterations of amino acids during the evolutionary process was also determined, with the score ranging from 43.667 (aa position 1202–1203) to 106.667 (aa position 5), suggesting a potential mutability impact on FYCO1. The three-dimensional model proposed FYCO1 as a disordered protein with some ordered regions at the N- and C-terminal areas. In domain-dependent analysis, out of a total of 19 prominently truncated mutations, 10 are present in the coiled-coil domain (Gln736Term lying in this region), two in the GOLD domain, one in the LIR domain, and one in between the FYVE and LIR domains of the currently unidentified domain. A previous functional elucidation of FYCO1 showed that the Znf domain intermingled with the RUN and GOLD domains in cytoskeleton proteins. The Clustal W program used to determine FYCO1 conservation showed 81% conservation between dogs, cows, and humans and 78% conservation between mice and humans [14,32]. These higher conservation rates are an indication of the extensive functional links of the protein.

Previous in silico studies also found two main effects of different *FYCO1* mutations (truncation and deletion) on protein conformation. They influence the formation of the Znf domain, which is highly necessary for the functioning of the FYCO1 protein. On the other hand, the deletion of the *FYCO1* gene without protein truncation causes regulatory changes; however, it is mostly involved in the loss of protein conformation and development of overall protein compactness due to the causative *FYCO1* mutation [14]. These results suggest the importance and crucial role of FYCO1 in acting as an adapter molecule for autophagy. The Znf domain of the FYCO1 protein interacts with nucleic acid or proteins, and its binding capacities are based on the domain sequence and its appropriate adaptation [33,34].

In our study, the STRING analysis of FYCO1 showed interaction potential with two members of the microtubule-associated protein 3 (MAP1LC3) family, MAP1LC3A and MAP1LC3B, both with functions in autophagy and autophagosome biogenesis. The other FYCO1 interaction is with RAB family members functioning in vesicle transport, such as RAB7B and RAB7A associated with Charcot–Marie–Tooth type 2 neuropathies and RAB40AL. FYCO1 also has interaction potential with CCZ1B involved in vacuolar transport, ZFYVE27 (the spastic paraplegia gene), CXCR6, and SASS6 involved in centriole duplication [35,36].

The association between the *FYCO1* mutation and autophagy in cataract development was established earlier [21]. In knockout mice, the lack of/deficiency in FYCO1 may lead to autophagosome deposition around the nucleus. Therefore, the transparency of the lens seems to be conserved via autophagy involving the synthesis of double-membranous autophagosomes that engulf the impaired cellular constituent and then degradation by lysosomal activities [37]. The scarcity of the Znf domain can provoke transport disruption, thus resulting in vesicle deposition. Although the mechanism of autophagy has been extensively studied for its role in disease processes, its association with CC is not yet clear and is debatable. On the other hand, autophagy may play an important role in the management of intracellular components. Therefore, its influence on age-related and congenital cataract progression requires extensive future research [38].

## 5. Conclusions

A previously reported *FYCO1*, c.2206C>T; p.Gln736Term variant was found in homozygous configuration in consanguineous families. Structural and functional characterization predicted that the mutation affects the coiled-coil domain of the FYCO1 protein. A three-dimensional model suggested that FYCO1 is a disordered protein. Protein–protein interaction studies of FYCO1 demonstrated its functions in autophagy and autophagosome biogenesis. It has been reflected that homozygous *FYCO1* mutations are a common cause of congenital cataracts in Pakistani families. It is suggested that establishing the precise and quick diagnosis of CC is of vital importance for affected individuals and their families as it expedites the basis of genetic counselling. It supports the integrative group to assist appropriately in child growth during a precarious stage, enables the congenital families with early diagnosis, and provides guidance to set the future strategies.

## Figures and Tables

**Table 1:** Physiological parameters of FYCO1.

Characteristic	Value/Symbol
Molecular weight	166982.63
Theoretical PI	4.86
Formula	C_7173_H_11580_N_2086_O_2388_S_54_
Total number of atoms	23281
Estimated half-life	30 h
Aliphatic index	79.42
Grand average of hydropathicity (GRAVY)	−0.737
Instability index	53.79

## Data Availability

All the available data included in this study can be found in the text of the paper.

## Supplementary Materials

The following supporting information can be downloaded at: https://www.mdpi.com/article/10.3390/life13081788/s1. Figure S1. Amino acid conservation pattern of FYCO1 predicted with the aid of ConSurf. The degree of conservation is indicated by color intensity, with highly conserved residues shown in maroon color, evolving sites with an average frequency in white, and variable sites in turquoise color. e: An exposed residue; b: a buried residue; f: a predicted functional residue (highly conserved and exposed); and s: a predicted structural residue (highly conserved and buried). Figure S2. Different physiochemical properties of FYCO1 were predicted with the aid of Protscale. X-axis indicates amino acid sequence from N- to C-terminal while Y-axis indicates scores computed by each algorithm, bulkiness; hydrophobicity; polarity; relative mutability.

## References

[1] Khokhar S.K., Pillay G., Dhull C., Agarwal E., Mahabir M., Aggarwal P. (2017). Pediatric cataract. Indian J. Ophthalmol..

[2] Wu X., Long E., Lin H., Liu Y. (2016). Prevalence and epidemiological characteristics of congenital cataract: A systematic review and meta-analysis. Sci. Rep..

[3] Sheeladevi S., Lawrenson J.G., Fielder A.R., Suttle C.M. (2016). Global prevalence of childhood cataract: A systematic review. Eye.

[4] Shiels A., Hejtmancik J.F. (2021). Inherited cataracts: Genetic mechanisms and pathways new and old. Exp. Eye Res..

[5] Reis L.M., Semina E.V. (2019). Genetic landscape of isolated pediatric cataracts: Extreme heterogeneity and variable inheritance patterns within genes. Hum. Genet..

[6] Şekeroğlu H., Utine G.E. (2021). Congenital Cataract and Its Genetics: The Era of Next-Generation Sequencing. Turk. J. Ophthalmol..

[7] Shoshany N., Hejtmancik F., Shiels A., Datiles M.B. (2020). Congenital and hereditary cataracts: Epidemiology and genetics. Pediatric Cataract Surgery and IOL Implantation: A Case-Based Guide.

[8] Iqbal H., Khan S.Y., Zhou L., Irum B., Ali M., Ahmed M.R., Shahzad M., Ali M.H., Naeem M.A., Riazuddin S. (2020). Mutations in FYCO1 identified in families with congenital cataracts. Mol. Vis..

[9] Dineen B., Bourne R.R., Jadoon Z., Shah S.P., Khan M.A., Foster A., Gilbert C.E., Khan M.D., Pakistan National Eye Survey Study Group (2007). Causes of blindness and visual impairment in Pakistan. The Pakistan national blindness and visual impairment survey. Br. J. Ophthalmol..

[10] Rahi J.S., Dezateux C. (2000). Congenital and infantile cataract in the United Kingdom: Underlying or associated factors. British Congenital Cataract Interest Group. Invest. Ophthalmol. Vis. Sci..

[11] Wasnik H., Gandhi R., Patil N., Behera R., Golait A., Patel T., Baruah T. (2021). A comprehensive review of molecular biology and genetics of cataract. Int. J. Res. Appl. Sci. Biotec..

[12] Hansen L., Mikkelsen A., Nürnberg P., Nürnberg G., Anjum I., Eiberg H., Rosenberg T. (2009). Comprehensive mutational screening in a cohort of Danish families with hereditary congenital cataract. Invest. Ophthalmol. Vis. Sci..

[13] Al-Badran R.A., Al-Badran A.I., Mabudi H., Neissi M., Mohammadi-Asl J. (2022). Detection of an FYCO1 nonsense mutation in an affected patient with autosomal recessive cataract (CTRCT18): A case report. Egyp. J. Med. Hum. Genet..

[14] Saleem R.S., Siddiqui S.N., Irshad S., Ashraf N.M., Hamid A., Khan M.A.U., Khan M.I., Micheal S. (2022). Targeted gene sequencing of FYCO1 identified a novel mutation in a Pakistani family for autosomal recessive congenital cataract. Mol. Genet. Genomic. Med..

[15] Barashkov N.A., Konovalov F.A., Borisova T.V., Teryutin F.M., Solovyev A.V., Pshennikova V.G., Sapojnikova N.V., Vychuzhina L.S., Romanov G.P., Gotovtsev N.N. (2021). Autosomal recessive cataract (CTRCT18) in the Yakut population isolate of Eastern Siberia: A novel founder variant in the FYCO1 gene. Eur. J. Hum. Genet..

[16] Chen J., Wang Q., Cabrera P.E., Zhong Z., Sun W., Jiao X., Chen Y., Govindarajan G., Naeem M.A., Khan S.N. (2017). Molecular Genetic Analysis of Pakistani Families with Autosomal Recessive Congenital Cataracts by Homozygosity Screening. Invest. Ophthalmol. Vis. Sci..

[17] Chen J., Ma Z., Jiao X., Fariss R., Kantorow W.L., Kantorow M., Pras E., Frydman M., Pras E., Riazuddin S. (2011). Mutations in FYCO1 cause autosomal-recessive congenital cataracts. Am. J. Hum. Genet..

[18] Li J., Chen X., Yan Y., Yao K. (2020). Molecular genetics of congenital cataracts. Exp. Eye Res..

[19] Abouzeid H., Helmy G., El Sada M., Sherif M., Yacoub M.H., Boisset G., Favez T., Schorderet D.F. (2012). FYCO1 mutation hotspot in congenital cataract. Invest. Ophthalmol. Vis. Sci..

[20] Nieto-Torres J.L., Shanahan S.L., Chassefeyre R., Chaiamarit T., Zaretski S., Landeras-Bueno S., Verhelle A., Encalada S.E., Hansen M. (2021). LC3B phosphorylation regulates FYCO1 binding and directional transport of autophagosomes. Curr. Biol..

[21] Pankiv S., Alemu E.A., Brech A., Bruun J.A., Lamark T., Overvatn A., Bjørkøy G., Johansen T. (2010). FYCO1 is a Rab7 effector that binds to LC3 and PI3P to mediate microtubule plus end-directed vesicle transport. J. Cell Biol..

[22] Shakil M., Harlalka G.V., Ali S., Lin S., D’Atri I., Hussain S., Nasir A., Shahzad M.A., Ullah M.I., Self J.E. (2019). Tyrosinase (TYR) gene sequencing and literature review reveals recurrent mutations and multiple population founder gene mutations as causative of oculocutaneous albinism (OCA) in Pakistani families. Eye.

[23] Kozlowski L.P., Bujnicki J.M. (2012). MetaDisorder: A meta-server for the prediction of intrinsic disorder in proteins. BMC Bioinform..

[24] Piovesan D., Tabaro F., Paladin L., Necci M., Micetic I., Camilloni C., Davey N., Dosztányi Z., Mészáros B., Monzon A.M. (2018). MobiDB 3.0: More annotations for intrinsic disorder, conformational diversity and interactions in proteins. Nucleic Acids. Res..

[25] Xue B., Oldfield C.J., Dunker A.K., Uversky V.N. (2009). CDF it all: Consensus prediction of intrinsically disordered proteins based on various cumulative distribution functions. FEBS. Lett..

[26] Ashkenazy H., Abadi S., Martz E., Chay O., Mayrose I., Pupko T., Ben-Tal N. (2016). ConSurf 2016: An improved methodology to estimate and visualize evolutionary conservation in macromolecules. Nucleic Acids. Res..

[27] Garnier J., Gibrat J.F., Robson B. (1996). GOR method for predicting protein secondary structure from amino acid sequence. Methods Enzymol..

[28] Cole C., Barber J.D., Barton G.J. (2008). The Jpred 3 secondary structure prediction server. Nucleic Acids. Res..

[29] Du Z., Su H., Wang W., Ye L., Wei H., Peng Z., Anishchenko I., Baker D., Yang J. (2021). The trRosetta server for fast and accurate protein structure prediction. Nat. Protoc..

[30] Szklarczyk D., Morris J.H., Cook H., Kuhn M., Wyder S., Simonovic M., Santos A., Doncheva N.T., Roth A., Bork P. (2017). The STRING database in 2017: Quality-controlled protein-protein association networks, made broadly accessible. Nucleic Acids. Res..

[31] Gillespie R.L., O’Sullivan J., Ashworth J., Bhaskar S., Williams S., Biswas S., Kehdi E., Ramsden S.C., Clayton-Smith J., Black G.C. (2014). Personalized diagnosis and management of congenital cataract by next-generation sequencing. Ophthalmology.

[32] Marchler-Bauer A., Anderson J.B., Chitsaz F., Derbyshire M.K., DeWeese-Scott C., Fong J.H., Geer L.Y., Geer R.C., Gonzales N.R., Gwadz M. (2009). CDD: Specific functional annotation with the Conserved Domain Database. Nucleic Acids. Res..

[33] Olsvik H.L., Lamark T., Takagi K., Larsen K.B., Evjen G., Øvervatn A., Mizushima T., Johansen T. (2015). FYCO1 Contains a C-terminally Extended, LC3A/B-preferring LC3-interacting Region (LIR) Motif Required for Efficient Maturation of Autophagosomes during Basal Autophagy. J. Biol. Chem..

[34] Johansen T., Lamark T. (2011). Selective autophagy mediated by autophagic adapter proteins. Autophagy.

[35] Wang Z., Miao G., Xue X., Guo X., Yuan C., Wang Z., Zhang G., Chen Y., Feng D., Hu J. (2016). The Vici Syndrome Protein EPG5 Is a Rab7 Effector that Determines the Fusion Specificity of Autophagosomes with Late Endosomes/Lysosomes. Mol. Cell.

[36] Suzuki H., Tabata K., Morita E., Kawasaki M., Kato R., Dobson R.C., Yoshimori T., Wakatsuki S. (2014). Structural basis of the autophagy-related LC3/Atg13 LIR complex: Recognition and interaction mechanism. Structure.

[37] Yang J., Yan R., Roy A., Xu D., Poisson J., Zhang Y. (2015). The I-TASSER Suite: Protein structure and function prediction. Nat. Methods.

[38] Morishita H., Mizushima N. (2016). Autophagy in the lens. Exp. Eye Res..

