# Supplementary material for: Identification and Functional Characterization of Mutation in FYCO1 in Families with Congenital Cataract
Source: Life (Basel). 2023 Aug 21;13(8):1788. doi: 10.3390/life13081788 (PMC10456301; doi:10.3390/life13081788)
Supplement: Supplementary file 1 [file life-13-01788-s001.zip › life-2523930-supplementary.pdf]

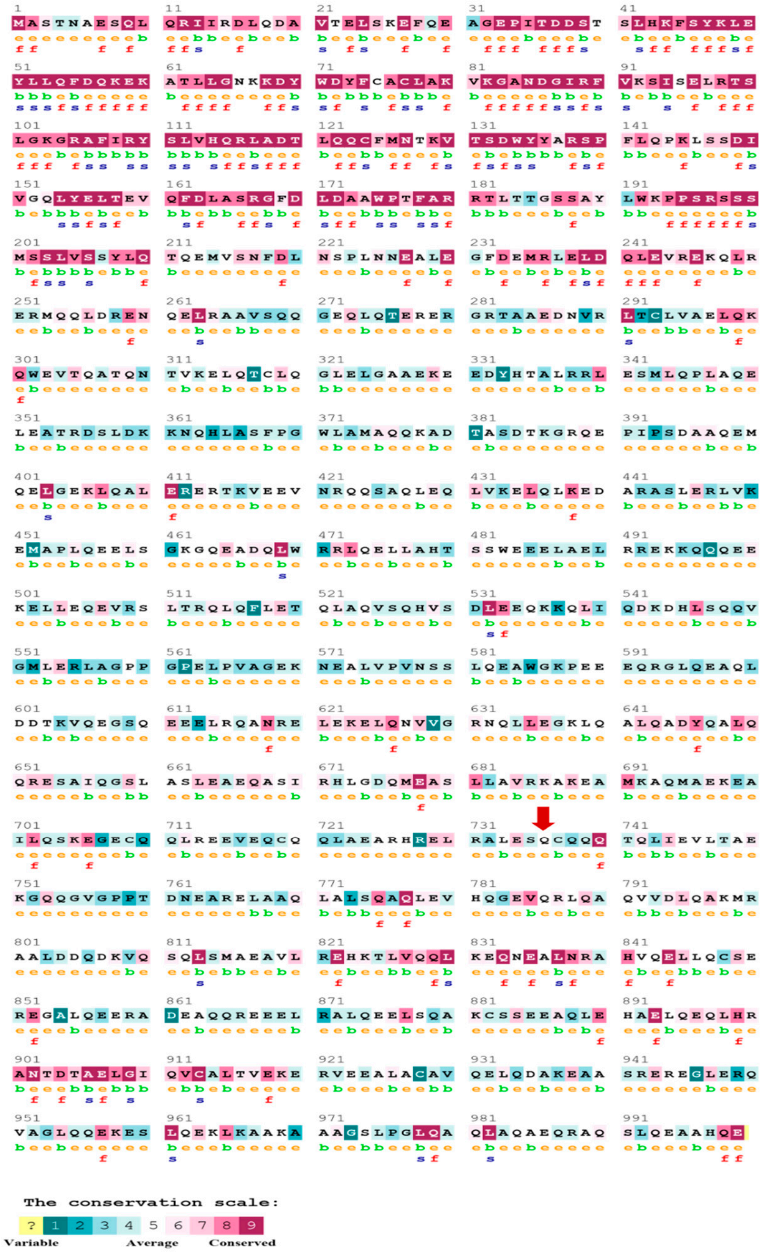

Supplementary Figure S1. Amino acid conservation pattern of FYCO1 predicted with the aid of ConSurf. The degree of conservation is indicated by color intensity, with highly conserved residues shown in maroon color, evolving sites with an average frequency in white, and variable sites in turquoise color. e: An exposed residue; b: a buried residue; f: a predicted functional residue (highly conserved and exposed); and s: a predicted structural residue (highly conserved and buried).

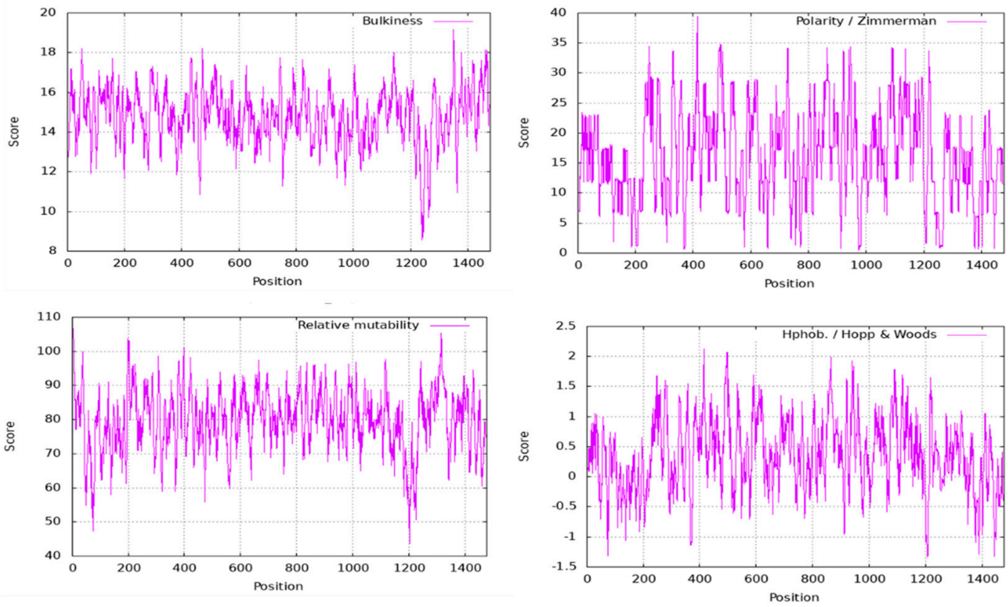

Supplementary Figure S2. Different physiochemical properties of FYCO1 were predicted with the aid of Protscale. X-axis indicates amino acid sequence from N- to C-terminal while Y-axis indicates scores computed by each algorithm, bulkiness; hydrophobicity; polarity; relative mutability.
